# Psychometric evaluation of the Swedish version of the 30-item endometriosis health profile (EHP-30)

**DOI:** 10.1186/s12905-020-01067-6

**Published:** 2020-09-14

**Authors:** Hanna Grundström, Anna Rauden, Per Wikman, Matts Olovsson

**Affiliations:** 1 Department of Women’s and Children’s Health, Uppsala University, Uppsala, Sweden; 2 Department of Obstetrics and Gynaecology in Norrköping, and Department of Biomedical and Clinical Sciences, Linköping University, Linköping, Sweden; 3 Department of Women’s Health, County Council of Gävleborg, Gävle, Sweden

**Keywords:** Endometriosis, EHP-30, The 30-item endometriosis health profile, Validity, Reliability, Quality of life

## Abstract

**Background:**

The 30-Item Endometriosis Health Profile (EHP-30) is a specific instrument measuring quality of life among women with endometriosis. Although the Swedish version of EHP-30 is widely used in research and clinical settings, it has not yet been evaluated psychometrically. Ensuring validity and reliability is of most importance when using translated instruments. Therefore, the aim of the study was to evaluate the psychometric properties of the Swedish version of the EHP-30.

**Methods:**

This study was conducted at a Swedish referral university hospital specializing in endometriosis. Data collection was performed in January 2013. The EHP-30 was sent to 369 randomly selected women with a laparoscopy-verified endometriosis diagnosis. The psychometric evaluation included evaluation of data completeness, score distributions, floor and ceiling effects, internal consistency, factor analysis and test-retest reliability.

**Results:**

Out of the 211 women with endometriosis who answered the questionnaire, 128 were native Swedish speakers who had experienced symptoms of endometriosis during the past 4 weeks, and were included in the psychometric evaluation. Data completeness was 99.5%. The highest median score was found in the Control and Powerlessness subscale, and lowest in Pain. Distributions towards ill health were found in all subscales except for the pain subscale, but there were no noteworthy floor or ceiling effects. Internal consistency was good (Cronbach’s α 0.83–0.96). Factor analysis could roughly confirm three of the five subscales. The test-rest analysis showed good reliability. Scores were systematically lower during the second measurement.

**Conclusions:**

We conclude that the Swedish version of EHP-30 is a valid and reliable instrument to measure health-related quality of life in women with endometriosis. It is understandable, acceptable and usable and can be recommended for use in clinical daily routines and for research purposes.

## Background

Endometriosis is a gynecological disease appearing in approximately every tenth woman of reproductive age. It is characterized by growth of endometrial cells outside the uterine cavity [1]. The etiology of endometriosis is in dispute, and theories on the pathogenesis of endometriosis propose that a combination immunological, hormonal, genetic and epigenetic factor factors may be involved development of the disease [2–4]. The most common symptoms are dysmenorrhea, dyspareunia and non-menstrual pelvic pain, lack of energy and infertility [1], often in combination with urinary or gastro-intestinal problems [5]. The disease often impairs women’s mental, physical, social and psychosexual well-being [6–9] and usually has a negative effect on health-related quality of life (HRQL) [10–12].

HRQL is a multi-dimensional concept which can be difficult to define. It incorporates those aspects of physical, mental and social life that may be associated with a disease or its treatment [13]. The negative impact of endometriosis upon HRQL is well documented [10, 14–17]. However, most studies used generic questionnaires, for example Short Form-36 (SF-36), which correlates poorly with pain intensity [18, 19]. Another limitation associated with generic instruments is that there are different conceptual frameworks, scales, and measurements used, which may limit the possibility to compare results and draw conclusions from several studies [14]. Furthermore, problems that may be unique to endometriosis, such as sexual difficulties or infertility, are not always addressed by generic questionnaires [20].

Therefore, the need for endometriosis-specific instruments has been raised. A specific instrument could lead to more accurate measurements of clinical outcomes, and consequently make meaningful changes to women’s lives. In the early 2000s, an endometriosis-specific instrument, the 30-item Endometriosis Health Profile (EHP-30), was developed from patient interviews and is presently the most reliable and most thoroughly validated questionnaire for HRQL measurement of women with endometriosis [21, 22]. The core questionnaire includes 30 items, and the modular questionnaire comprises 23 more specific items that may not apply to all women with endometriosis. The use of EHP-30 is recommended by the American Society for Reproductive Medicine, the European Society for Human Reproduction and Embryology [23] and the National Board of Health and Welfare in Sweden [24].

In Sweden, EHP-30 has been used in both research and in clinical settings. The Swedish translation has gone through cross-cultural adaption, which resulted in a minor change of wording in one question [25]. However, to the best of our knowledge, the Swedish version of the core questionnaire EHP-30 has not yet been psychometrically tested. Thus, the aim of the present study was to evaluate the psychometric properties of the Swedish version of EHP-30.

## Methods

### Study design and data collection

The EHP-30 questionnaires were sent by mail to 369 randomly selected women with a laparoscopy-verified endometriosis diagnosis who had visited the National Endometriosis Center in Uppsala, Sweden during the years 2007–2010. The questionnaires were sent out during the period 23–28 January 2013. All participants also completed demographic questions regarding age, marital status, parity, highest completed education, native language and main occupation. The study group was asked about year at symptom onset and year at receiving the endometriosis diagnosis and whether they had experienced symptoms of endometriosis in the last 4 weeks. If the questionnaires were not returned within 3 weeks, another questionnaire was sent together with a reminder.

The first 150 women who returned their questionnaires were immediately sent one more questionnaire in order to evaluate test-retest reliability.

The sample size calculation was based on Terwee et al. who recommend at least 50 participants for test-retest reliability and a subject–item ratio of between 4 and 10 (with a minimum number of 100) to ensure stability of the factor analysis [26]. In this study, a minimum sample size of 120 was required related to the 30 items in the questionnaire. All analyses were conducted on individuals who reported symptoms in the last 4 weeks and whose native language was Swedish.

The study was approved by the regional ethics committee in Uppsala in 2010-06-29 (Reg. no. 2011/220).

### The endometriosis health profile − 30

The EHP-30 contains 30 questions divided into five subcategories. These categories address problem-areas applicable to many women with endometriosis: pain (questions 1 to 11), control and powerlessness (questions 12 to 17), emotional wellbeing (questions 18 to 23), social support (questions 24 to 27) and self-image (questions 28 to 30). The questions are presented to ask how often in the last 4 weeks the respondent has experienced difficulties in a certain aspect: never, rarely, sometimes, often or always (five-point Likert scale (0–4)). Each scale is translated into a score ranging from 0 (best possible health status) to 100 (worst possible health status) by dividing the subscale scores by the maximum possible raw score within the subscale and multiplying it by 100 [21].

### Psychometric evaluation

a) Data completeness: The acceptance of the questionnaire was determined by calculating the response rates. For items with missing data, mean imputation was performed.

b) Descriptive statistics, score distributions and floor and ceiling effects: data were presented using mean, standard deviation, median, 25th and 75th percentiles and coefficient of skewness. Floor or ceiling effects were considered to be notable if more than 15% of respondents achieved the lowest or highest possible scores, respectively [26].

c) Internal consistency: Cronbach’s α coefficient was used to evaluate internal consistency. Values above 0.70 were considered to indicate that individual items in a subscale were sufficiently correlated to be summarized into the same scale [27].

d) Factor analysis: To assess the underlying structure of the questionnaire principal component analysis using varimax rotation was performed. Loadings above 0.40 were reported.

e) Test-retest reliability: reliability over time was examined using the test-retest method. Women who reported a change in their self-experienced health compared with answers to their first questionnaire, were omitted from the test-retest analysis. Intra class correlation (ICC) with a two way random, single measures, absolute agreement model was used to assess reliability in repeated measurements. Estimated coefficients below 0.75 have been interpreted as evidence for a poor reliability [28]. Score differences between the two measurements were assessed using rank sum tests. *P*-values < 0.05 were considered statistically significant.

All analyses were conducted using R.3.6.1.

## Results

### Participants

A total of 211 women with endometriosis answered the questionnaire, which gave a primary response rate of 57%. Of these, 128 were native Swedish speakers who had experienced symptoms of endometriosis during the past 4 weeks. They were included in the psychometric evaluation, resulting in a subject–item ratio of 4:1. Demographic characteristics of the study participants are shown in Table 1.

**Table 1:** Demographic characteristics of the study participants (*n* = 128)

Variable	n(%) or mean ± SD
**Age**	38 ± 8
**Number of children**	
No children	68 (53%)
One child	26 (20%)
Two children or more	34 (27%)
**Marital status**	
Single	39 (30%)
In a relationship	89 (70%)
**Highest level of education**	
Compulsory school	6 (5%)
Secondary education	51 (40%)
University education	70 (55%)
**Occupation**	
Working/studying full-time	63 (49%)
Working/studying part-time	30 (23%)
On sick-leave	17 (13%)
Other	18 (14%)
**Years from symptom onset to diagnosis**	8 ± 13

### Data completeness, score distributions, and floor and ceiling effects

**Fig. 1:**
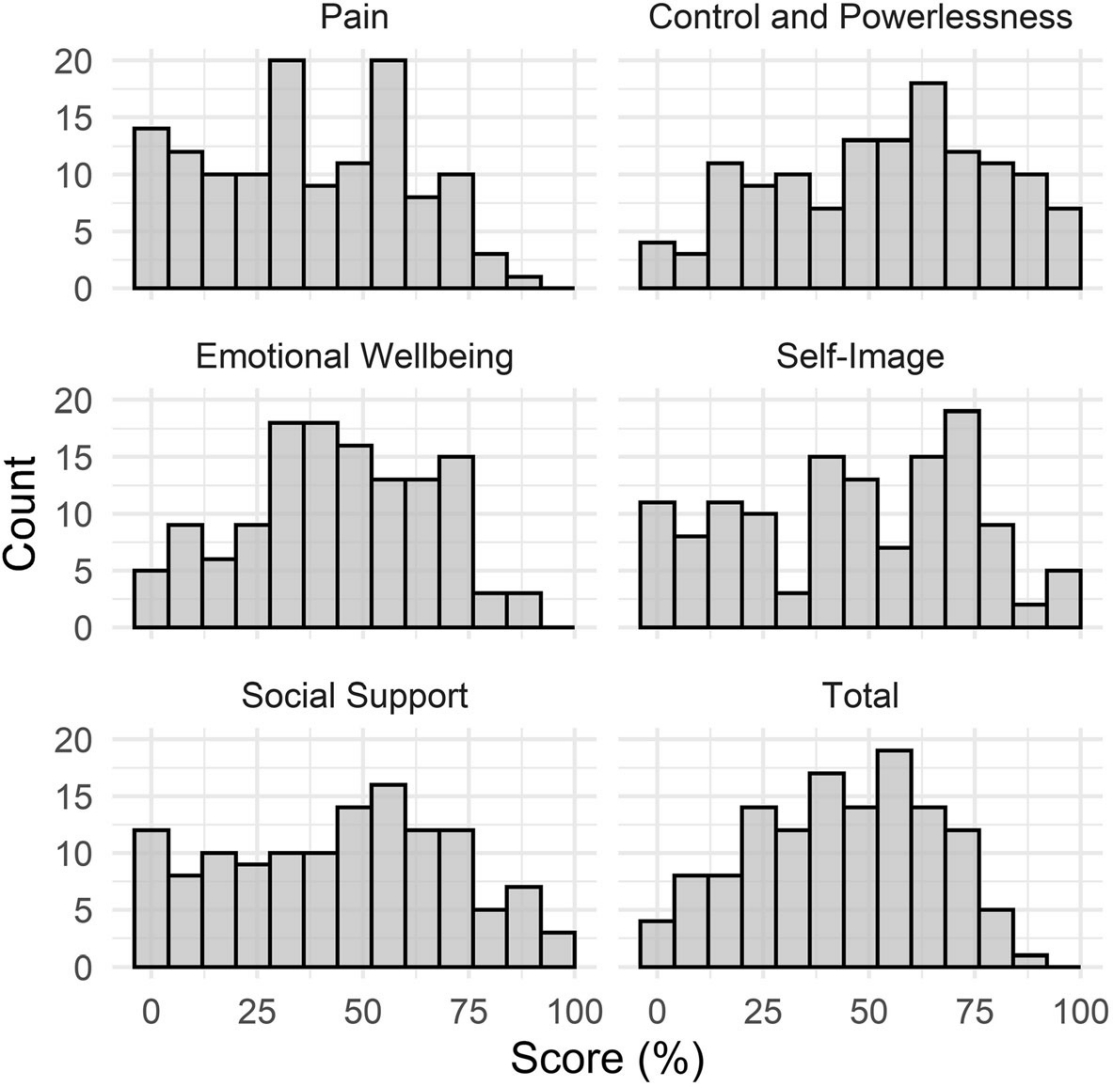
Score distribution as percentages of the maximum attainable score per subscale and in total

The descriptive statistics including score distributions and floor and ceiling effects for the five subscales of EHP-30 are presented in Table 2, while score distributions as percentages of the maximum attainable score per subscale and in total are presented in Fig. 1.

**Table 2:** Score distributions, skewness, floor and ceiling effects and internal consistency for EHP-30

Subscale of EHP-30	Median	25th percentile	75th percentile	Coefficient of skewness	Floor effect	Ceiling effect	Cronbachs alpha
Pain	34	16	57	0.03	0.08	0.00	0.96
Control & Powerlessness	58	33	75	−0.22	0.03	0.02	0.92
Emotional Wellbeing	42	29	62	−0.08	0.04	0.00	0.91
Social Support	50	25	69	−0.18	0.09	0.02	0.88
Self-Image	50	25	67	−0.05	0.09	0.02	0.83
Total	44	26	60	−0.15	0.01	0.00	0.97

Nearly 100% of data completeness was achieved. Out of the 128 participants, 122 (95.3%) answered all questions, four participants (3.1%) answered 29 questions and two participants (1.6%) answered 23 questions. This resulted in a total data completeness of 99.5%. The highest median score was found in the Control and Powerlessness subscale (58), while Pain had the lowest (34). Negatively skewed distributions towards ill health were found in all subscales except for the pain subscale, for which a small positive distribution was observed. Control and Powerlessness had the most negative distribution (− 0.22). No notable floor or ceiling effects were found.

### Internal consistency

Cronbach’s α coefficient ranged from 0.83 to 0.96 for the subscales, which indicated good internal consistency (Table 2).

### Factor analysis

Factor analysis could roughly confirm three of the five previously established [15] subscales of the questionnaire (Table 3). Of the five extracted principal components, factors one through to three respectively correspond to the scales Pain, Emotional Wellbeing (EW), and Control and Powerlessness (C&P).

**Table 3:** Factor analysis: factor loadings for EHP-30

Item	Loading factor 1	Loading factor 2	Loading factor 3	Loading factor 4	Loading factor 5
*Pain scale*					
1. Unable to go to social events	0.73				
2. Unable to do jobs around the home	0.75				
3. Found it difficult to stand	0.80				
4. Found it difficult to sit	0.75				
5. Found it difficult to walk	0.83				
6. Found it difficult to exercise/leisure activities	0.80				
7. Lost appetite/unable to eat	0.72				
8. Unable to sleep properly	0.65				
9. Had to go to bed/lie down	0.78				
10. Unable to do the things you want to do	0.83				
11. Felt unable to cope with the pain	0.71				
*Control and Powerlessness scale*					
1. Generally felt uncomfortable	0.44		0.41		0.43
2. Symptoms not getting better	0.43		0.69		
3. Not able to control symptoms			0.66		
4. Unable to forget symptoms			0.79		
5. Symptoms ruling life			0.76		
6. Symptoms taking away life			0.66		
*Emotional Wellbeing scale*					
1. Felt depressed		0.68			
2. Felt weepy/tearful		0.73			
3. Felt miserable		0.56			0.46
4. Had mood swings		0.82			
5. Felt bad or short-tempered		0.78			
6. Felt violent or aggressive		0.77			
*Social Support scale*					
1. Unable to tell people how you feel		0.47		0.58	
2. Felt others do not understand				0.65	
3. Others think you are moaning			0.40	0.57	
4. Felt alone		0.49		0.46	
*Self-Image scale*					
1. Cannot wear clothes you choose				0.76	
2. Appearance has been affected				0.78	
3. Lacked confidence		0.46		0.63	

Items belonging to subscales Social Support (Soc) and Self-image (Self) both loaded on the fourth factor thus the fifth factor could not specifically be attributed to any particular subscale. Factors one through to four accounted for 94% of the total variance.

Several items loaded on more than one factor, but the majority of items loaded higher on their “own” factor. Items on the Pain subscale loaded only on their factor, while item C&P1 (Generally felt unwell) loaded on three factors, including its own (Control and Powerlessness, Pain and the fifth factor). Item EW3 (Felt miserable) loaded on Emotional wellbeing and on the fifth factor.

The seven items in Social Support and in Self-image all loaded on the fourth factor (the combined Social Support and Self-image component), but four of the items also had loadings on other factors: item Soc1 (Unable to tell people how you feel) loaded on Emotional Wellbeing, item Soc3 (Others think you are moaning) on Control and Powerlessness, item Soc4 (Felt alone) and Self3 (Lacked confidence) on Emotional wellbeing.

### Test-retest reliability

The response rate of the second questionnaire to assess test-retest reliability was 47% with 70 symptomatic women returning the questionnaire. Out of these, 28 women reported a change in health and were excluded from the test-rest analysis, resulting in 42 questionnaires for the test-retest analysis. Participants answered the second questionnaire in median 9 days after the first one. The ICC for agreement ranged from 0.82 to 0.86, indicating good reliability. Scores were systematically lower during the second measurement in most subscales, but the differences were not statically significant (Table 4).

**Table 4:** Subscale median scores, ICC, ICC confidence intervals and *p*-value for the test-retest questionnaires

Subscale of EHP-30		First questionnaire (***n*** = 42)	Second questionnaire (***n*** = 42)	ICC	ICC confidence intervals	***p***-value
**Pain**	Median	31	32	0.85	0.76–0.91	0.90
	25th percentile	12	21			
	75th percentile	55	55			
**Control &** **Powerlessness**	Median	52	48	0.82	0.72–0.88	0.30
	25th percentile	28	30			
	75th percentile	75	66			
**Emotional** **Wellbeing**	Median	44	35	0.83	0.73–0.89	0.90
	25th percentile	18	21			
	75th percentile	61	50			
**Social Support**	Median	50	50	0.85	0.78–0.91	0.51
	25th percentile	25	25			
	75th percentile	67	67			
**Self-Image**	Median	50	47	0.86	0.79–0.91	0.52
	25th percentile	13	19			
	75th percentile	63	67			

## Discussion

In the present study we used four criteria recommended in guidelines for psychometric evaluation [26] to assess the psychometric properties of the Swedish version of EHP-30: score distributions, internal consistency, factor analysis, and test-retest reliability. In general, our results indicate high validity and reliability of the Swedish version of EHP-30. Further criteria such as responsiveness of the Swedish version have been assessed with acceptable results [29]. Content validity and construct validity were assessed by the authors of the original version [21, 30].

In our data nearly 100% completeness was achieved, which suggests that the questionnaire is understandable and accessible. However, the majority of the participants had a university degree (55%), which may have contributed to the high level of data completeness. In the average Swedish population, 28% have a higher education [31].

The Control and Powerlessness subscale showed the highest median score, which is concordant with validation studies from The Netherlands, the UK, France, the US, Norway, China and Australia, [22, 30, 32–36]. This indicates that loss of control and power has a severely negative impact on HRQL in women with endometriosis. Hence, empowerment and patient participation could be important to highlight for improvement work within endometriosis care.

Our data showed good internal consistency (0.83–0.96), which is high compared with previous research [22, 30, 32–35, 37, 38]. Three of the subscales exceeded α 0.90, indicating suitability for measuring outcomes at an individual level [27].

The factor analysis confirmed a four-factor model for the questionnaire, in contrast to the five subscales established in the original version [21]. Five factors were found in the Dutch, French, Chinese, Portuguese and Persian versions [22, 32, 35, 37, 38], while the Norwegian version was three-factored [34]. In our data, several items loaded on more than one scale. Only the Pain scale had no items loading on other scales. Two of the items in Control and Powerlessness loaded on the Pain scale. In other studies the Pain and Control and Powerlessness scales were the most common to overlap [32, 34, 37], and in the UK version, the overlap was complete [30]. This suggests that there is a strong association between lack of control and power and the pain experience, which should be considered when encountering these women. The last seven items were all loaded to the same factor (the combined Social Support and Self-image component), which was also seen in the Norwegian version [34]. This indicates that the last seven questions are partly measuring the same construct, which is important to bear in mind when interpreting the results. Thereby, wide-reaching conclusions from the last two dimensions should be drawn with caution.

Test-retest reliability was high (ICC 0.82–0.86), and the lowest ICC was higher than in previous studies [32, 34, 39]. The women scored slightly lower the second time, but the differences between the two measurements were not statically significant and are not likely to represent a clinical relevant difference [28].

There are some limitations to this study. Firstly, recruiting participants from an endometriosis referral center may have resulted in a selected study group with an over representation of women with severe symptoms. The problems with recruitment of a representative sample of participants with endometriosis has been raised before, and is a well-known challenge in endometriosis research [16]. Most studies on validation of EHP-30 included women from referral centers or patient organizations, leading to a possible over representation of participants with severe disease in all studies, and thereby the results could be comparable [22, 32, 34–38]. Secondly, there was a relatively low participation rate. Out of the other validation studies, the Norwegian and Dutch studies used comparable sampling. While Verket et al. had a lower participation rate (42%) [34], van de Burgt et al. had a higher rate (76%) [22]. In our study, unfortunately the sample size felt short of the desired *n* = 50 for the test-retest. This may have resulted in broader confidence intervals and less power, but is not likely to have any major impact on the results or bias the results in any considerable aspect. Thirdly, criterion validity was not addressed [26].

## Conclusions

In summary, we found high data completeness, low floor and ceiling effects, good internal consistency and excellent test-retest reliability. Our factor analysis roughly confirmed three of the five factors of the questionnaire, with an overlapping of the Social support and Self-image subscales. Overall, we conclude that the Swedish version of EHP-30 is a valid, reliable, understandable, acceptable and usable instrument that can be used to measure HRQL in daily clinical practice and in research. Further research could focus on the modular questions, which are not yet validated in a Swedish context.

## Data Availability

The datasets used and/or analyzed during the current study available from the corresponding author on reasonable request.

## Abbreviations

HRQL: Health-related quality of life
EHP-30: The Endometriosis Health Profile − 30
ICC: Intra class correlation
EW: Emotional wellbeing
C&P: Control and Powerlessness
Soc: Social Support
Self: Self-image

## References

[1] Dunselman GA, Vermeulen N, Becker C, Calhaz-Jorge C, D'Hooghe T, De Bie B (2014). ESHRE guideline: management of women with endometriosis. Hum Reprod.

[2] Vetvicka V, Laganà AS, Salmeri FM, Triolo O, Palmara VI, Vitale SG (2016). Regulation of apoptotic pathways during endometriosis: from the molecular basis to the future perspectives. Arch Gynecol Obstet.

[3] Laganà AS, Vitale SG, Salmeri FM, Triolo O, Frangež HB, Vrtačnik-Bokal E (2017). Unus pro omnibus, omnes pro uno: a novel, evidence-based, unifying theory for the pathogenesis of endometriosis. Med Hypotheses.

[4] Vitale SG, Capriglione S, Peterlunger I, La Rosa VL, Vitagliano A, Noventa M. The role of oxidative stress and membrane transport systems during endometriosis: a fresh look at a busy corner. Oxidative Med Cell Longev. 2018;7924021. 10.1155/2018/7924021 PMC5883985 29743986

[5] Schomacker ML, Hansen KE, Ramlau-Hansen CH, Forman A (2018). Is endometriosis associated with irritable bowel syndrome? A cross-sectional study. Eur J Obstet Gynecol Reprod Biol.

[6] Culley L, Law C, Hudson N, Denny E, Mitchell H, Baumgarten M (2013). The social and psychological impact of endometriosis on women’s lives: a critical narrative review. Hum Reprod Update.

[7] Young K, Fisher J, Kirkman M (2015). Women’s experiences of endometriosis: a systematic review and synthesis of qualitative research. J Fam Plan Reprod Heal Care.

[8] Vitale SG, La Rosa VL, Rapisarda AMC, Laganà AS (2017). Endometriosis and infertility: the impact on quality of life and mental health. J Endometr Pelvic Pain Disord..

[9] La Rosa VL, De Franciscis P, Barra F, Schiattarella A, Tropea A, Tesarik J (2020). Sexuality in women with endometriosis: a critical narrative review. Minerva Med.

[10] Jia S-Z, Leng J-H, Shi J-H, Sun P-R, Lang J-H (2012). Health-related quality of life in women with endometriosis: a systematic review. J Ovarian Res.

[11] Bourdel N, Chauvet P, Billone V, Douridas G, Fauconnier A (2019). Systematic review of quality of life measures in patients with endometriosis. PLoS One.

[12] La Rosa VL, De Franciscis P, Barra F, Schiattarella A, Török P, Shah M. Quality of life in women with endometriosis: a narrative overview. Minerva Med 2020;111(1):68–78. 10.23736/S0026-4806.19.06298-0 31755667

[13] Guyatt GH, Feeny DH, Patrick DL (1993). Measuring health-related quality of life. Ann Intern Med.

[14] Nnoaham K, Hummelshoj L, Webster P (2011). Europe PMC funders group impact of endometriosis on quality of life and work productivity: a multicenter study across ten countries. Fertil Steril.

[15] Simoens S, Dunselman G, Dirksen C, Hummelshoj L, Bokor A, Brandes I (2012). The burden of endometriosis: costs and quality of life of women with endometriosis and treated in referral centres. Hum Reprod.

[16] De Graaff AA, D’Hooghe T, Dunselman GA, Dirksen C, Hummelshoj L, Simoens S (2013). The significant effect of endometriosis on physical, mental and social wellbeing: results from an international cross-sectional survey. Hum Reprod.

[17] Grundström H, Gerdle B, Alehagen S, Berterö C, Arendt-Nielsen L, Kjølhede P (2019). Reduced pain thresholds and signs of sensitization in women with persistent pelvic pain and suspected endometriosis. Acta Obstet Gynecol Scand.

[18] Abbott JA (2003). The effects and effectiveness of laparoscopic excision of endometriosis: a prospective study with 2-5 year follow-up. Hum Reprod.

[19] Marques A, Bahamondes L, Aldrighi JM, Fernandes LF, Augusto K, Brilhante A (2004). Quality of life in Brazilian women with endometriosis assessed through a medical outcome questionnaire. J Reprod Med.

[20] Neelakantan D, Omojole F, Clark TJ, Gupta J, Kahn K (2004). Quality of life instruments in studies of chronic pelvic pain: a systematic review. J Obstet Gynaecol.

[21] Jones G, Kennedy S, Barnard A, Wong J, Jenkinson C (2001). Development of an endometriosis quality-of-life instrument: the endometriosis health Profile-30. Obstet Gynecol.

[22] van de Burgt TJM, Hendriks JCM, Kluivers KB (2011). Quality of life in endometriosis: evaluation of the Dutch-version endometriosis health profile–30 (EHP-30). Fertil Steril.

[23] Vincent K, Kennedy S, Stratton P (2010). Pain scoring in endometriosis: entry criteria and outcome measures for clinical trials. Report from the art and science of endometriosis meeting. Fertil Steril.

[24] National Board of Health and Welfare (Socialstyrelsen). National Guidelines For Endometriosis Care (in Swedish). 2018. https://www.socialstyrelsen.se/globalassets/sharepoint-dokument/artikelkatalog/nationella-riktlinjer/2018-12-27.pdf Accessed 28 Feb 2020.

[25] Grundström H, Rauden A, Olovsson M. Cross-cultural adaptation of the Swedish version of endometriosis health Profile-30. J Obstet Gynecol. 2020. 10.1080/01443615.2019.1676215. 10.1080/01443615.2019.1676215 31909643

[26] Terwee CB, Bot SDM, de Boer MR, van der Windt D, Knol D, Dekker J (2007). Quality criteria were proposed for measurement properties of health status questionnaires. J Clin Epidemiol.

[27] Cronbach LJ, Warrington WG (1951). Time-limit tests: estimating their reliability and degree of speeding. Psychometrika..

[28] Koo TK, Li MY (2016). A guideline of selecting and reporting intraclass correlation coefficients for reliability research. J Chiropr Med.

[29] Wickstrom K, Spira J, Edelstam G (2017). Responsiveness of the endometriosis health Profile-30 questionnaire in a Swedish sample: an observational study. Clin Exp Obstet Gynecol.

[30] Jones G, Jenkinson C, Taylor N, Mills A, Kennedy S (2006). Measuring quality of life in women with endometriosis: tests of data quality, score reliability, response rate and scaling assumptions of the endometriosis health profile questionnaire. Hum Reprod.

[31] Statistics Sweden (Statistiska Centralbyrån) Higher education in Sweden (In Swedish) 2019. https://www.scb.se/hitta-statistik/sverige-i-siffror/utbildning-jobb-och-pengar/utbildningsnivan-i-sverige/ Accessed 4 Dec 2019.

[32] Chauvet P, Auclair C, Mourgues C, Canis M, Gerbaud L, Bourdel N (2017). Psychometric properties of the French version of the endometriosis health Profile-30, a health-related quality of life instrument. J Gynecol Obstet Hum Reprod.

[33] Jenkinson C, Kennedy S, Jones G (2008). Evaluation of the American version of the 30-item endometriosis health profile (EHP-30). Qual Life Res.

[34] Verket NJ, Andersen MH, Sandvik L, Tanbo TG, Qvigstad E (2018). Lack of cross-cultural validity of the endometriosis health Profile-30. J Endometr Pelvic Pain Disord.

[35] Jia S-Z, Leng J-H, Sun P-R, Lang J-H (2013). Translation and psychometric evaluation of the simplified Chinese-version endometriosis health Profile-30. Hum Reprod.

[36] Khong S-U, Lam A, Luscombe G (2010). Is the 30-item endometriosis health profile (EHP-30) suitable as a self-report health status instrument for clinical trials?. Fertil Steril.

[37] Nogueira-Silva C, Costa P, Martins C, Barata S, Alho C, Calhaz-Jorge C (2015). Validation of the Portuguese Version of EHP-30 (The Endometriosis Health Profile-30). Acta Med Port.

[38] Nojomi M, Bijari B, Akhbari R, Kashanian M (2011). The assessment of reliability and validity of Persian version of the endometriosis health profile (EHP-30). Iran J Med Sci.

[39] van de Burgt TJM, Kluivers KB, Hendriks JCM (2013). Responsiveness of the Dutch endometriosis health Profile-30 (EHP-30) questionnaire. Eur J Obstet Gynecol Reprod Biol.

